# Medical and Physical Intelligence

**Published:** 1821-10

**Authors:** 


### Medical and Physical Intelligence.

MEDICO-CHIRURGICAL *Society of Edinburgh.*—IT is with pleasure that we announce the formation of a Medico-Chirurgical Society in Edinburgh. The Society is formed upon the model of the Medico-Chirurgical Society of London, and has in view precisely similar objects. Most of the medical Professors in the University, and many of the most respectable practitioners in the city, have co-operated in its formation. Dr. DUNCAN, sen. has been elected its first president; its sittings commence in the approaching winter-season.

In addition to ordinary and honorary members, provision is made for the admission of corresponding members; and it is hoped that many, in almost every part of the world, and such especially as retain a grateful recollection of the advantages they derived from their Alma Mater, will not be backward in supplying interesting communications.

Communications may be transmitted to the President of the Society, or to either of the Secretaries, according to the following addresses:— Dr. W. P. ALISON, No. 44, Heriot-row, Edinburgh; Dr. ROBERT HAMILTON, No. 3, Northumberland-street, Edinburgh.

*Case of Poisoning by Arsenic, successfully treated.*—A VERY singular case occurred lately, in which the recovery from the poisonous effects of arsenic was obtained, and the patient saved, in a much shorter period than usually happens under similar circumstances. We understand that the management was conducted by Mr. HUME, of Long-Acre, who ascribes his success chiefly to an incessant administration of magnesia and opium. The importance of the subject will, we hope, induce Mr. Hume to send us the particulars of this interesting case, which must be very acceptable to the profession and to the community at large.

*Institution for the Cure of Injuries and Diseases of the Eyes, at Winchester.*—A SMALL number of medical practitioners in the city of Winchester having set on foot a plan for the establishment of an Eye Infirmary, Mr. MAYO, who is one of the surgeons to the County Hospital, addressed a letter to the chairman, in July last, in reply to the observations contained in the prospectus of the proposed charity, respecting the supposed inability of a general hospital in affording relief to that particular class of diseases in so eminent a degree as might be done at an institution solely appropriated to that purpose. Mr. Mayo states, that—

"During the ten years in which I have been surgeon to the hospital, a large share of the ophthalmic patients have come under my care, and I have had considerable success in their cure, both by operations and constitutional treatment; and I also beg to state, that, some years ago, having somewhat of a similar object in view to that which my colleagues now propose, I made inquiries of my professional brethren about the county for cases of cataract, and other diseases of the eye, which would admit of relief by operation, but that I found by far too small a number to justify the recommendation of such a scheme to public patronage.

"A very laudable zeal has, no doubt, prompted my colleagues to solicit the attention of the public to the present plan; but, as a servant of the county, equally zealous for the promotion of every thing calculated to relieve the infirmities of the lower orders, I have no hesitation in expressing my confidence that our hospital can afford every relief to persons afflicted with diseases of the eyes in this county; and that, with very little alteration in its establishment, it might embrace every advantage which my colleagues anticipate from a distinct institution.

"During the last eight years, there have not been more than 160 eye-cases sent to the county-hospital, making an annual average of twenty, of which the proportion of in-patients is twelve, and of out-patients eight; and the proportion of eye-cases to all other cases admitted, is about one to fifty. The smallness of this proportion will suffice to show that there could be no great difficulty or expence in providing for their occasional select accommodation, as I have above proposed."

The plan has, however, it appears, been carried into effect; and the *Hampshire Chronicle*, of Monday, September the 17th, we are happy to find, has the following paragraph, proving the correctness of the expectations of those who suggested the propriety of forming a separate institution.

"Since the opening of the Eye Infirmary, in this city, a period of only two months, we understand that fifty-three patients have been registered on the books; three of whom (one born blind,) have submitted to operations for cataract."

---

*Mexican Flora.*—At the last anniversary sitting of the Helvetic Society of Natural Science, M. de Candolle presented to the Society a *Flora of Mexico*, consisting of 1740 leaves, and forming thirteen large folio volumes. The following account of this work is given in the *Morgenblatt*, published at Stutgard:—MM. Sesse, Mocino, and Cervantes, had travelled over New Spain, with the view of collecting a Mexican Flora. They made a drawing of each plant on the spot where they found it. M. Mocino had returned to Madrid, in order to have the drawings thus obtained engraved, when the first troubles in

Spain obliged him to seek refuge with his Flora at Montpelier. M. de Candolle, who was then at Montpelier, became acquainted with M. Mocino, and assisted him for eighteen months in arranging systematically his numerous collection. M. de Candolle afterwards went from Montpelier to Geneva, and M. Mocino gave him the Flora along with him, that he might one day send it forth to the world. The new aspect of affairs in Spain having induced M. Mocino, however, to return to his native country, he wrote lately to M. de Candolle, requesting to have the Flora back. The French naturalist, unwilling to run the chance of losing all trace of so valuable a treasure, immediately requested some friends to copy part of the rarest drawings for him. No sooner was this known in Geneva, than numbers of persons of both sexes offered their services; and, in the end, every person capable of managing a crayon or a pencil was occupied with the Mexican Flora. They worked with such zeal, the ladies especially, that in the short space of eight days there was not a single drawing remaining to copy.—*Philosophical Mag.*

---

*The Unicorn.*—Mr. Campbell (the missionary) has kindly favoured us with the following description of the head of a very singular animal which he has just brought from the interior of Africa. We also have had an opportunity of seeing it, and fully agree with Mr. Campbell that the animal itself must have answered the description of the *Reem* or *Unicorn*, which is frequently mentioned in Scripture.

"The animal," says Mr. Campbell, "was killed by my Hottentots, in the Mashow county, near the city of Mashow, about two hundred miles N.E. of New Lattakoo, to westward of Delagoa Bay. My Hottentots, never having seen or heard of an animal with *one* horn of so great a length, cut off its head, and brought it bleeding to me upon the back of an ox. From its great weight, and being about twelve hundred miles from the Cape of Good Hope, I was obliged to reduce it by cutting off the under jaw. The Hottentots cut up the rest of the animal for food, which, with the help of the natives, they brought on the backs of oxen to Mashow.

"The horn, which is nearly black, is exactly three feet long, projecting from the forehead about nine or ten inches above the nose. From the nose to the ears measured three feet. There is a small horny projection of about eight inches immediately behind the great horn, designed for keeping fast or steady whatever is penetrated by the great horn. There is neither hair nor wool on the skin, which is the colour of brown snuff.

"The animal was well known to the natives. It is a species of the rhinoceros; but, if I may judge of its bulk from the size of its head, it must have been much larger than any of the seven rhinoceroses which my party shot, one of which measured eleven feet from the tip of the nose to the root of the tail.

"The skull and horn excited great curiosity at the Cape. Most were of opinion that it was all we should have for the unicorn.

"An animal, the size of a horse, which the fancied unicorn is supposed to be, would not answer the description of the unicorn given by

Job, chap. xxxix. verse 9 *et seq.*, but in every part of that description this animal exactly answers to it."—*Idem.*

*Academical Prize Question.*—The Society of Sciences and Arts of Utrecht has announced the following question for competition:—"Are there characteristic signs sufficient to distinguish always with certainty the true cancers from other maladies which resemble it? If so, what are these signs? Ought this malady to be considered as the effect of an indisposition of the whole body, or as only local? If it is to be considered as an indisposition of the whole body, can external remedies, whether amputation, or the remedy applied by the religious of the convent of Rus, or the corrosive remedies, especially arsenic, contribute to the cure or the alleviation of the malady? or ought they to be considered as all equally hurtful? When the malady has not yet the characteristic signs of true cancer, but when there is reason to fear it may become so, and when it may as yet be considered as a local evil, what external remedies may then be applied with sound hope of success? and what are those which should be considered as hurtful?"

*Cathartine, the active Principle of Senna.*—MM. J. L. Lassaigne and H. Fenuelle, in examining senna, obtained from it a particular principle, called by them cathartine. A decoction of the leaves was made, and, after being filtered, was precipitated by acetate of lead. The precipitate collected was diffused through water, and sulphuretted hydrogen passed through it. The liquor filtered was evaporated to dryness, and digested in alcohol, and the alcohol solution then evaporated to dryness. It contained acetate of potassa, which was separated by alcohol acidulated by sulphuric acid; then, filtering to separate the sulphate of potassa insoluble in this fluid, precipitating the excess of sulphuric acid by acetate of lead, decomposing this latter salt by sulphuretted hydrogen, filtering again and evaporating to dryness, a substance was obtained, which was considered the purgative principle of senna.

This substance is uncrystallizable, of a reddish yellow colour, of a particular smell, a bitter and nauseous taste. It is soluble in alcohol and water in all proportions; insoluble in ether. Its extract becomes moist in the air. It purges in very small doses.—*Annales de Chimie*, xvi. p. 20.

*Piperin, or the active Principle of Pepper.*—Piperin is a new vegetable principle, extracted from black pepper, by Mr. Pelletier. To obtain it, black pepper was digested in alcohol repeatedly, and the solution evaporated, until a fatty resinous matter was left. This, on being washed in warm water, was left of a good green colour, and had a hot and burning taste; it dissolved readily in alcohol, and less readily in sulphuric ether; concentrated sulphuric acid gave it a fine scarlet colour. A solution of this substance in hot alcohol, being left for some days, deposited a number of small crystals. These were purified by repeated solution and crystallization in alcohol and ether, and from the mother liquors fresh portions were obtained, which, on purification, were like the first. It is to be remarked, that the pepper taste they possessed when impure, gradually left them as they became more and more pure; so that the white crystals scarcely had any taste, while it seemed to accumulate in the fatty matter, as the crystalline portion was separated from it; and also that, the purer the crystals, the finer the tint produced in them by sulphuric acid. The fatty matter left also reddened by sulphuric acid; but it is a question whether it would do so when pure.

The crystalline matter forms colourless four-sided prisms, with single inclined terminations; they have scarcely any taste. Boiling water dissolves a small portion, but it is insoluble in cold water; it is very soluble in alcohol, less so in ether; it is soluble in acetic acid, and crystallizes from it in feathery crystals. Weak sulphuric, nitric, and muriatic acids, do not dissolve or act on it; the strong acids decompose it. Strong sulphuric acid gives it a blood-red colour, which disappears on adding water; the substance does not seem altered if the acid has not remained long on it. Muriatic acid acts in the same way, producing a deep-yellow colour. Nitric acid makes it greenish yellow, orange, and then red; the ultimate action of the acid produces oxalic acid, and yellow bitter principle. It melts at about 212°. Destructive distillation converts it into water, acetic acid, oil, and carburetted hydrogen gas: no ammonia is formed. Oxide of copper converts it into carbonic acid and water. After comparing this substance with other vegetable principles, particularly with resins, Mr. Pelletier concludes by considering it a peculiar substance, and names it Piperin.

The fatty matter, left after extracting the piperin, is solid at a temperature near 32°, but liquefies at a slight heat. It has an extremely bitter and acrid taste; it is very slightly volatile, and tends rather to decompose than rise in vapour; that which passes over is not so piquant and acrid as the undistilled part, but is more balsamic. It dissolves easily in alcohol and ether, and unites to fatty bodies; and, with the exception of its taste, does not differ from them. From the result of the distillation, it may be considered as composed of two oils: one, volatile and balsamic; the other, more fixed, and containing the acridness of the pepper.

Finally, Mr. Pelletier finds in pepper:—1, piperin; 2, a very acrid concrete oil; 3, a volatile balsamic oil; 4, a gummy coloured matter; 5, an extractive principle; 6, malic and tartaric acids; 7, starch; 8, bassorine; 9, lignin; 10, earthy and alkaline salts. He concludes, also, that there is no vegetable alkali in pepper; that the crystalline substance of pepper is a peculiar body; that pepper owes its taste to an oil but little volatile; and that a strong similarity exists between common pepper and cubebs, as illustrated by the analysis of Mr. Vauquelin, of the latter compared with the former.—*Idem.* p. 337.

---

*Internal Use of Iodine.*—Dr. Gimelle, first assistant-surgeon of the military hospital of the Royal Guards, at Paris, has published, in the Bulletin of the Medical Society of Emulation, the history of several cases of bronchocele, and a few of herpetic eruptions, in which he administered the iodine, either internally or externally, with complete success.—*See the Number for August,* 1821.

### METEOROLOGICAL JOURNAL.

By Messrs. William Harris and Co. 50, Holborn, London.

*From August* 20 *to September* 19, *inclusive.*

| Day of Month. | Moon. | Rain gauge | THERM. | | | BAROM. | | DeLuc's Hygro. | | WIND. | | ATMOSPHERIC VARIATION. | | |
|---|---|---|---|---|---|---|---|---|---|---|---|---|---|---|
| Aug | | | | | | | | | | | | | | |
| 20 | ☽ | | 63 | 74 | 60 | 30·19 | 30·21 | 53 | 54 | E | ESE | Fine | | |
| 21 | | | 63 | 76 | 63 | 30·20 | 30·17 | 54 | 51 | E | ENE | Fine | | |
| 22 | | | 65 | 75 | 63 | 30·16 | 30·14 | 62 | 61 | ESE | ESE | Cloudy | Fine | |
| 23 | | | 65 | 74 | 62 | 30·08 | 30·01 | 62 | 61 | ENE | E | Cloudy | Fine | |
| 24 | | | 63 | 75 | 60 | 29·95 | 29·93 | 61 | 61 | E | E | Cloudy | Fine | |
| 25 | | | 68 | 47 | 60 | 29·97 | 29·95 | 60 | 61 | SW | SW | Fine | | |
| 26 | | | 63 | 67 | 59 | 30·01 | 30·00 | 60 | 61 | ENE | E | Rain | Fine | Cloud. |
| 27 | ● | | 60 | 60 | 56 | 30·07 | 30·12 | 58 | 59 | ESE | SE | Cloudy | | |
| 28 | | ·15 | 59 | 63 | 50 | 29·95 | 29·88 | 58 | 60 | E | E | Rain | Cloud. | Rain |
| 29 | | ·22 | 53 | 60 | 55 | 29·81 | 29·74 | 60 | 60 | SE | SE | Rain | | |
| 30 | | | 57 | 65 | 58 | 29·73 | 29·71 | 57 | 61 | SW | SSW | Fine | Sho'ry | Rain |
| 31 | | ·25 | 59 | 68 | 60 | 29·74 | 29·81 | 60 | 63 | W | WNW | Fine | Rain | Th.&Li. |
| Sep. | | | | | | | | | | | | | | |
| 1 | | ·13 | 60 | 66 | 61 | 29·90 | 30·01 | 62 | 60 | NE | NW | Rain | | Cloud. |
| 2 | | | 64 | 68 | 60 | 30·02 | 30·05 | 59 | 59 | W | W | Fine | | |
| 3 | | ·05 | 63 | 71 | 62 | 29·97 | 29·91 | 63 | 59 | SW | SW | Showery | Fine | |
| 4 | ☾ | ·11 | 65 | 70 | 59 | 29·83 | 29·77 | 60 | 60 | WSW | WSW | Showery | | Fine |
| 5 | | | 62 | 71 | 60 | 29·90 | 30·00 | 58 | 59 | SW | S | Fine | | |
| 6 | | | 61 | 68 | 57 | 29·91 | 29·85 | 60 | 60 | S | SSE | Cloudy | Fine | Cloud. |
| 7 | | | 60 | 68 | 55 | 29·70 | 29·57 | 60 | 60 | S | SSW | Cloudy | | |
| 8 | | ·14 | 59 | 65 | 53 | 29·61 | 29·75 | 60 | 60 | W var. | WSW | Rain | Cloud. | Fine |
| 9 | | ·16 | 55 | 61 | 49 | 29·69 | 29·64 | 61 | 61 | W var. | W var. | Showery | | |
| 10 | | ·19 | 53 | 60 | 51 | 29·74 | 29·88 | 61 | 59 | W var. | W var. | Showery | | Fine |
| 11 | ○ | | 57 | 65 | 56 | 30·00 | 30·00 | 58 | 58 | W var. | W var. | Fine | | |
| 12 | | ·14 | 59 | 63 | 55 | 29·58 | 29·85 | 58 | 58 | W var. | W var. | Showery | | Fine |
| 13 | | ·13 | 58 | 67 | 53 | 29·91 | 29·98 | 58 | 60 | W | WNW | Showery | | Rain |
| 14 | | ·25 | 57 | 65 | 60 | 30·09 | 30·15 | 61 | 60 | SE | NE | Rain | Cloud. | |
| 15 | | | 61 | 67 | 57 | 30·19 | 30·24 | 60 | 61 | NE | N | Fog | Fine | Foggy |
| 16 | | | 59 | 67 | 60 | 30·17 | 30·20 | 60 | 64 | W | W | Cloudy | Fine | Cloud. |
| 17 | | | 61 | 69 | 58 | 30·21 | 30·12 | 58 | 58 | WNW | W | Fine | | |
| 18 | ☽ | | 63 | 71 | 57 | 29·95 | 29·88 | 57 | 55 | W | W | Fine | | |
| 19 | | | 61 | 70 | 69 | 29·95 | 30·02 | 55 | 55 | W | NW | Fine | | |

The quantity of rain fallen in the month of August, is 1 inch and 67-100ths.

